# Metformin targets intestinal immune system signaling pathways in a high-fat diet-induced mouse model of obesity and insulin resistance

**DOI:** 10.3389/fendo.2023.1232143

**Published:** 2023-09-19

**Authors:** Monta Brīvība, Laila Silamiķele, Ineta Kalniņa, Ivars Silamiķelis, Līga Birzniece, Laura Ansone, Lauma Jagare, Ilze Elbere, Jānis Kloviņš

**Affiliations:** Latvian Biomedical Research and Study Centre, Riga, Latvia

**Keywords:** metformin, NF-kappa B signaling, humoral immune responses, high-fat diet, intestinal transcriptome

## Abstract

**Introduction:**

Research findings of the past decade have highlighted the gut as the main site of action of the oral antihyperglycemic agent metformin despite its pharmacological role in the liver. Extensive evidence supports metformin’s modulatory effect on the composition and function of gut microbiota, nevertheless, the underlying mechanisms of the host responses remain elusive. Our study aimed to evaluate metformin-induced alterations in the intestinal transcriptome profiles at different metabolic states.

**Methods:**

The high-fat diet-induced mouse model of obesity and insulin resistance of both sexes was developed in a randomized block experiment and bulk RNA-Seq of the ileum tissue was the method of choice for comparative transcriptional profiling after metformin intervention for ten weeks.

**Results:**

We found a prominent transcriptional effect of the diet itself with comparatively fewer genes responding to metformin intervention. The overrepresentation of immune-related genes was observed, including pronounced metformin-induced upregulation of immunoglobulin heavy-chain variable region coding *Ighv1-7* gene in both high-fat diet and control diet-fed animals. Moreover, we provide evidence of the downregulation NF-kappa B signaling pathway in the small intestine of both obese and insulin-resistant animals as well as control animals after metformin treatment. Finally, our data pinpoint the gut microbiota as a crucial component in the metformin-mediated downregulation of NF-kappa B signaling evidenced by a positive correlation between the *Rel* and *Rela* gene expression levels and abundances of *Parabacteroides distasonis*, *Bacteroides spp*., and *Lactobacillus spp*. in the gut microbiota of the same animals.

**Discussion:**

Our study supports the immunomodulatory effect of metformin in the ileum of obese and insulin-resistant C57BL/6N mice contributed by intestinal immunoglobulin responses, with a prominent emphasis on the downregulation of NF-kappa B signaling pathway, associated with alterations in the composition of the gut microbiome.

## 1 Introduction

In the management of diabetes mellitus, metformin (dimethyl-biguanide) is still considered the first-choice pharmacological treatment for most patients because of its relatively high efficacy, affordable price, and safety (1). Nevertheless, it is more often accepted to use other classes of agents with metformin as an early combination therapy since evidence shows that 21% of patients using metformin as a monotherapy eventually do not reach their glycemic goals, while up to 20% of patients do not tolerate the drug due to gastrointestinal adverse events (1–3).

The generally accepted mechanism of action of metformin is the activation of AMP-activated protein kinase as a result of inhibition of complex I with a subsequent improvement in hepatic insulin sensitivity and inhibition of hepatic gluconeogenesis (4). However, these assumptions have been challenged by multiple studies discovering the highly pleiotropic effect of the drug ranging from anti-aging properties (5) to anti-inflammatory effects (6) and recognition of the gastrointestinal tract as one of the main sites of action of the drug with a significant contribution of gut microbiota yet with no persuasive underlying mechanism clarified so far (7).

Over the past decade, much effort has been made to describe the modulatory effect of metformin on the composition and functions of gut microbiota in both human and animal studies, revealing metformin-induced alterations in the abundance of *Akkermansia* spp. and *Clostridium* spp., though with notable divergence among the hosts studied (8, 9). Meanwhile, the reports describing intestinal host responses show ambiguous results with distinct molecular targets and signaling pathways affected by the drug. Studies show that metformin improves glucagon-like peptide 1 (GLP‐1) secretion (10), targets the glucose-SGLT1 sensing by modifying microbiota of the upper small intestine of rodents (11), restores diet-induced overexpression of key genes for the transport of glucose and fatty acids in the small intestine of mice (12), and changes the expression of glycolytic genes such as *Slc2a1*, *Slc2a2*, and *Slc5a1* in the intestinal organoids (13). In addition to glucose homeostasis, several studies provide considerable evidence of the intestinal immunomodulatory effect of the drug including the ability of metformin to prevent high-fat diet-induced reduction of IgA-producing cells, increase the levels of fecal sIgA (14, 15), and even improve the tight junction of intestinal epithelial cells (16).

Since data describing gut-specific host responses to metformin therapy *in vivo* are lacking, we aimed to identify the intestinal molecular targets of metformin in mice at the state of obesity and insulin resistance by performing a global transcriptome analysis. To the best of our knowledge, this is the first study providing bulk RNA-Seq data of the distal part of the small intestine of high-fat diet-fed and metformin-treated C57BL/6N mice of both sexes with appropriate control arms.

## 2 Methods

### 2.1 Study design

**Figure 1:**
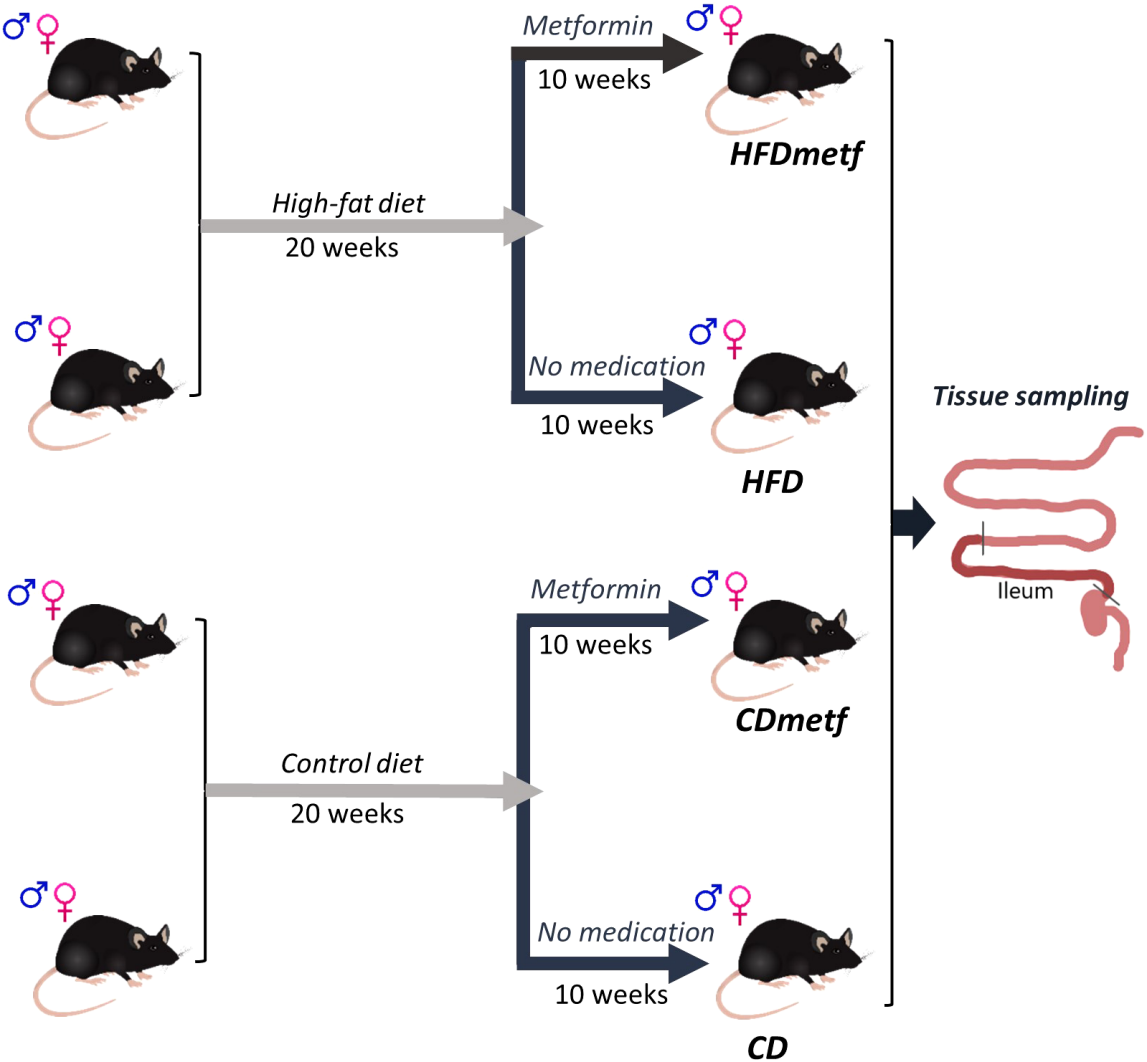
Schematic representation of the experimental design. At first, 72 four- to five-week-old C57BL/6N mice of both sexes were randomly divided into two groups (36 animals per group), where each study group received a different diet (either a high-fat diet or the control diet with a regular fat content) for 20 weeks. After the diet intervention, half of the animals representing each diet-related study group (*n* = 18) were randomly selected for the following metformin intervention in drinking water for 10 weeks. Equal animal sex distribution was repeatedly ensured during each randomization procedure, resulting in four different study arms: animals receiving a control diet only (CD), animals receiving a control diet followed by metformin intervention (CD_Metf), animals receiving a high-fat diet only (HFD), and animals receiving a high-fat diet followed by metformin therapy (HFD_Metf). At the end of the animal experiment, the tissue sample from the distal part of the small intestine (ileum) was collected from three males and three females representing each study arm (24 samples in total).

Since the animal experiment was conducted for simultaneous gut microbiome data acquisition, the study was designed as a randomized block experiment, where each study arm was represented by three experimental units in specific pathogen-free conditions. An animal cage with three animals in it was considered as one experimental unit of the study due to an inevitable exchange of the microbiota between different animals within one cage. Experimental units/cages were organized in three blocks (24 animals per block) with time as the only blocking factor and a 2-week-long time shift between each block resulting in a 4-week-long extension of the experiment in total. In total, 72 animals (36 males and 36 females) were involved in the study compiling 24 experimental units. Half of the animals (*n* = 36) were allocated to the high-fat diet intervention, while the rest of the animals (*n* = 36) received the control diet after the first randomization procedure. The 20-week-long diet intervention was followed by a 10-week-long metformin treatment to which half of the animals (*n* = 16) of each diet group were randomly subjected. An equal animal sex distribution was ensured by performing the randomization procedures in males and females separately. Finally, there were eight study groups developed with three experimental units (nine animals) in each group based on three different factors (sex, diet, and therapeutic intervention): 1) high-fat diet-fed (HFD-fed) male mice receiving metformin, 2) HFD-fed male mice with no metformin intervention, 3) HFD-fed female mice receiving metformin, 4) HFD-fed female mice with no metformin intervention, 5) control diet-fed (CD-fed) male mice receiving metformin, 6) CD-fed male mice with no metformin intervention, 7) CD-fed female mice receiving metformin, and 8) CD-fed female mice with no metformin intervention. Nevertheless, for the RNA-Seq analysis, only one animal from each cage was randomly selected, resulting in the intestinal transcriptome data set derived from six animals (three males and three females) of each of the treatment groups: high-fat diet-fed animals (HFD), high-fat diet-fed animals receiving metformin (HFDmetf), control diet-fed animals (CD), and control diet-fed animals receiving metformin (CDmetf), representing all three experimental blocks. A schematic representation of the experimental design is provided in **Figure 1**.

### 2.2 Animals

Four- to 5-week-old male and female C57BL/6N mice were obtained from the University of Tartu Laboratory Animal Centre. Animals of the same sex were housed in individually ventilated cages (Tecniplast, Milan, Italy) in groups of three. Mice were fed with either a high-fat diet (HFD: 60 kcal% fat, D12492, Research Diets, New Jersey, USA) or a control diet (CD: 10 kcal% fat, D12450J, Research Diets, New Jersey, USA) *ad libitum* depending on the experimental group assigned. The room temperature was controlled at 23°C ± 2°C with 55% humidity and a 12:12-h light–dark cycle.

### 2.3 Experimental procedures

For half of the animals, an obesity and insulin resistance phenotype was induced by HFD for 20 weeks. After week 20, metformin therapy was provided for randomly selected animals (half of both HFD-fed and CD-fed mice) with drinking water for 10 weeks in a concentration of 50 mg/kg body mass/day. The dose of metformin was chosen according to previous findings proving metformin dosage of 50 mg/kg/day as a clinically relevant dose retaining the property to improve hyperglycemia in high-fat diet-fed animals (17). The phenotype of experimental animals was monitored: body weight was measured once a week, and 6-h fasting plasma glucose was determined using an Accu-Chek Performa glucometer (Roche, Vienna, Austria) once in 2 weeks. In addition, at weeks 20 (before administration of metformin) and 30 (after 10 weeks of metformin therapy), fasting glucose and insulin levels were determined using the mouse glucose assay and mouse insulin ELISA kit (Crystal Chem, Zaandam, Netherlands) to calculate the homeostatic model assessment for insulin resistance (HOMA-IR) (18). Stool samples were collected from animals before cervical dislocation and analyzed using the Mouse Secretory Immunoglobulin A (sIgA) ELISA Kit (MyBioSource, USA) following the manufacturer’s instructions, and the absorbance was read at 450 nm. All the animals were euthanized by cervical dislocation without any other anesthesia to avoid potential bias of medications other than metformin. For tissue sampling from the distal part of the small intestine (ileum), one animal representing each experimental unit/cage was randomly selected (24 animals in total). Each tissue sample was stored at −80°C in 1.5 mL of RNAlater solution (Thermo Fisher Scientific, USA) until further processing.

### 2.4 Sample processing and next-generation sequencing

RNA extraction from intestinal tissue was performed using AllPrep DNA/RNA Mini Kit (QIAGEN, Germany) according to the manufacturer’s instructions. For rRNA depletion, 200 ng of total RNA was processed using MGIEasy rRNA depletion kit (MGI Tech Co. Ltd., China). Complementary DNA library preparation was performed with MGIEsy RNA Directional Library Prep Set (MGI Tech Co. Ltd., China). The quantity and quality of extracted RNA and prepared libraries were determined by Qubit Fluorometer (Thermo Fisher Scientific, USA) and Agilent 2100 Bioanalyzer system (Agilent, USA), respectively. The integrity of RNA was evaluated by RNA integrity number (RIN) within the Agilent 2100 Bioanalyzer system (Agilent, USA). Next-generation sequencing was conducted on the DNBSEQ-G400RS sequencing platform (MGI Tech Co. Ltd., China), following the manufacturer’s instructions. Since shotgun RNA sequencing is considered to be the most accurate method for quantification of the expression of individual transcripts and genes, additional methods for technical validation were not applied in this study (19).

Isolation of microbial DNA from stool samples collected after metformin intervention from the same animals and the following shotgun metagenomic sequencing was done as described before (9). Briefly, for the DNA extraction, FastDNA® Spin Kit for Soil (MP Biomedicals, USA) was used according to the manufacturer’s instructions. The DNA libraries were prepared with MGIEasy Universal DNA Library Prep Kit (MGI Tech Co. Ltd., China) adding 1% PhiX control and sequenced on the DNBSEQ-G400RS next-generation sequencing platform (MGI Tech Co. Ltd., China) using the DNBSEQ-G400RS High-throughput Sequencing Set (FCL PE100) (MGI Tech Co. Ltd., China). The desired sequencing depth was at least 20 M 100 bp paired-end sequencing reads per sample.

### 2.5 Data analysis

For the trimming of sequencing reads, a sliding window size of 5 and a quality threshold of 25 were applied in Trimmomatic 0.39. Sequencing reads had to have a minimum length of 30 bp and an average quality of 25 to be included in the subsequent analyses. The reads were mapped to the mouse reference genome GRCm38, and per-gene read counts were calculated with STAR 2.5.3a. The obtained read counts were then normalized using the trimmed mean of M-values implemented in Bioconductor package edgeR (v.3.24.3) in R (v.3.5.3). For the gene filtering, filterByExpr() function was applied, taking into account the library sizes and the experimental design (20). Differentially expressed genes (DEGs) were identified with limma package (v.3.38.3) and voom method using a linear model with weighted least squares for each gene (21). Both diet and therapy were defined as factors to evaluate their transcriptional effects, while the animal sex and sequencing block were considered covariates. DEGs were identified by comparing animals representing different experimental groups in four contrasts: HFD-fed animals against CD-fed animals with no metformin therapy applied in both groups (HFD vs. CD), HFD-fed animals against CD-fed animals with metformin therapy applied for both groups (HFDmetf vs. CDmetf), HFD-fed animals with metformin therapy against HFD-fed animals with no metformin therapy (HFDmetf vs. HFD), and finally, CD-fed animals with metformin therapy compared with CD-fed animals with no metformin therapy applied (CDmetf vs. CD). Multiple testing correction was implemented using the Benjamini–Hochberg procedure, and significant DEGs were determined using a false discovery rate (FDR) <0.05 cutoff (22).

Gene Ontology (GO) terms and Kyoto Encyclopedia of Genes and Genomes (KEGG) pathway analysis was performed using the online software Database for Annotation, Visualization and Integrated Discovery (DAVID) 6.8 applying a *P*-value threshold <0.05 (23, 24). Heatmaps were constructed with Matplotlib⁠ and SciPy⁠. Hierarchical clustering was performed with the average linkage method implemented in SciPy for the clustering of genes according to their differences in CPM values (25, 26). Cnetplot for GO terms was constructed using the clusterProfiler package in R (27).

For the correlation analysis, features (genes and taxonomies) with a median value of 100 reads were retained for further analyses. Read counts were normalized using a centered log-ratio (clr) transform using scikit-bio (0.5.8). Before clr transformation, imputation of zero values was performed with geometric Bayesian multiplicative replacement as implemented in R package zCompositions (1.4.0-1). Integration of metagenomic and transcriptomic data was performed with sparse partial least squares (sPLS) by selecting at most 30 variables from each contrast with mixomics (6.20.0). Pearson correlations of features were visualized as clustered image maps (heatmaps) using the complete linkage method as an agglomerative clustering algorithm and using Euclidean distances as a distance measure for both types of features. Feature stability was evaluated with 10-fold cross-validation and 100 repeats. Predictors with variable importance in projection value (VIP) >1 were considered relevant, while the frequency of occurrence >0.8 was accepted as stable.

For the calculation of sIgA concentrations, the interpolation was done in GraphPad Prism by using the sigmoidal 4PL model. The *p*-values for the comparison of HOMA-IR, body mass, glucose, insulin, and sIgA levels were calculated using the Kruskal–Wallis test, followed by Dunn’s test for pairwise comparisons with the Bonferroni adjustment, and visualized with the ggplot2 package implemented in R.

## 3 Results

### 3.1 Diet- and metformin-induced phenotypic alterations

In total, 24 animals were included in the study, with six animals representing each of the four treatment groups and equal gender distribution in each group (three males and three females). Four different contrasts were used in the statistical analysis of phenotypic as well as RNA-Seq data considering both the diet and metformin intervention (HFD vs. CD, HFDmetf vs. CDmetf, HFDmetf vs. HFD, CDmetf vs. CD); nevertheless, the greatest emphasis was placed on the contrasts reflecting the effect of metformin at the state of obesity and insulin resistance as well as the control phenotype (HFDmetf vs. HFD and CDmetf vs. CD).

**Figure 2:**
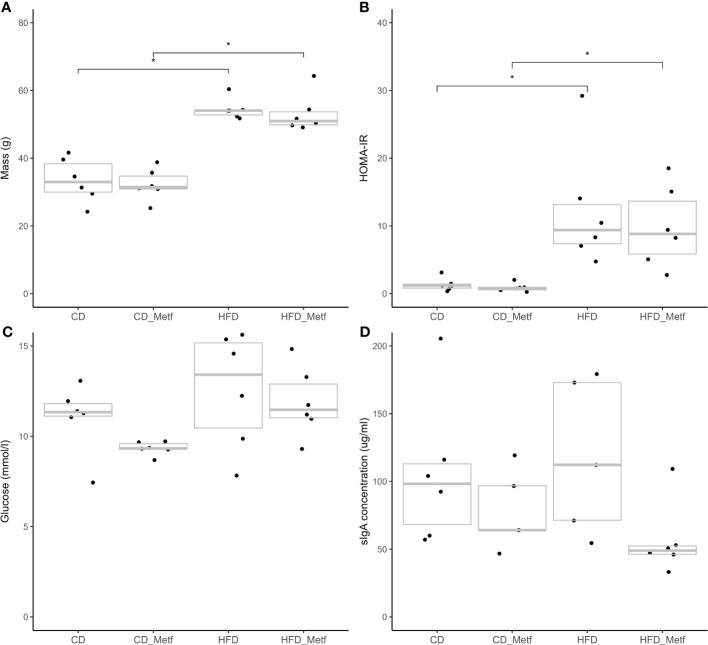
Jitter plots representing the phenotypic differences among animals of different treatment arms at the end of the study. Four different measurements are shown: **(A)** body mass, **(B)** homeostatic model assessment for insulin resistance, **(C)** blood glucose levels, and **(D)** secretory IgA levels. Each dot represents a single measurement for each animal with a gray crossbar indicating the median and IQR of the corresponding experimental group. Comparisons with statistically significant differences are marked with the asterisk above the square brackets (adjusted *P*-value threshold of 0.05). IQR, interquartile range; CD, control diet-fed animals; CD_Metf, control diet-fed animals receiving metformin; HFD, high-fat diet-fed animals; HFD_Metf, high-fat diet-fed animals receiving metformin.

To evaluate the intervention-induced phenotypic alterations, body mass and fecal sIgA levels were detected in addition to the HOMA-IR index, which was calculated for each animal based on fasting plasma glucose and insulin levels at the end of the experiment. Significantly higher body mass (HFD: median = 54.06 g, IQR = 52.38–54.26 g; HFDmetf: median = 50.96 g, IQR = 49.69–54.39 g; CD: median = 32.96 g, IQR = 29.56–39.60 g; CDmetf: median = 31.46 g, IQR = 30.86–35.69 g), insulin levels (HFD: median = 1,358.33 pmol/L, IQR = 652.05–1,918.17 pmol/L; HFDmetf: median = 1,072.35 pmol/L, IQR = 513.20–1,682.04 pmol/L; CD: median = 134.73 pmol/L, IQR = 78.65–174.68 pmol/L; CDmetf: median = 116.72 pmol/L, IQR = 78.83–134.70 pmol/L), and also HOMA-IR indexes (HFD: median = 9.39, IQR = 7.06–14.06; HFDmetf: median = 8.83, IQR = 5.06–15.07; CD: median = 1.21, IQR = 0.61–1.89; CDmetf: median = 0.82, IQR = 0.51–0.93) were detected in HFD-fed animals compared with CD-fed animals at the end of the experiment in all of the comparisons tested. The difference in blood glucose levels (HFD: median = 13.41 mmol/L, IQR = 9.87–15.37 mmol/L; HFDmetf: median = 11.47 mmol/L, IQR = 10.97–13.28 mmol/L; CD: median = 11.34 mmol/L, IQR = 11.06–11.95 mmol/L; CDmetf: median = 9.33 mmol/L, IQR = 9.27–9.67 mmol/L) did not reach statistical significance after adjusting the *p*-value. Similarly, although not reaching statistical significance, the body mass, HOMA-IR, glucose, and insulin levels tend to show lower levels in mice receiving metformin treatment against the untreated animals irrespective of the diet applied (**Figures 2A–C**, **Supplementary Table 5**).

According to the previously reported property of metformin to elevate sIgA levels in human stool, additional ELISA-based measurements of fecal sIgA were performed after metformin intervention, showing notable though not significant variability in different treatment arms (HFD: median = 112.25 μg/mL, IQR = 63.01–176.24 μg/mL; HFDmetf: median = 48.92 μg/mL, IQR = 42.74–67.02 μg/mL; CD: median = 98.30 μg/mL, IQR = 59.38–138.35 μg/mL; CDmetf: median = 64.03 μg/mL, IQR = 55.33–107.92 μg/mL) (**Figure 2D**, **Supplementary Table 5**). The data describing phenotypic variation among animals of different sexes are provided in **Supplementary Table 5** and **Supplementary Figure 7**).

### 3.2 Diet-induced alterations in transcriptome profiles of the distal part of the small intestine

The RNA-Seq approach was applied to comprehensively identify transcripts responding to the diet and/or metformin treatment in samples obtained from the distal part of the small intestine of the 24 animals. Next-generation sequencing provided a median of 18.1 million sequencing reads per sample (IQR = 68.4), where 73.9% (IQR = 20.4) of the reads were mapped to the reference genome, ensuring the identification of 55,536 unique mouse genes in total.

**Figure 3:**
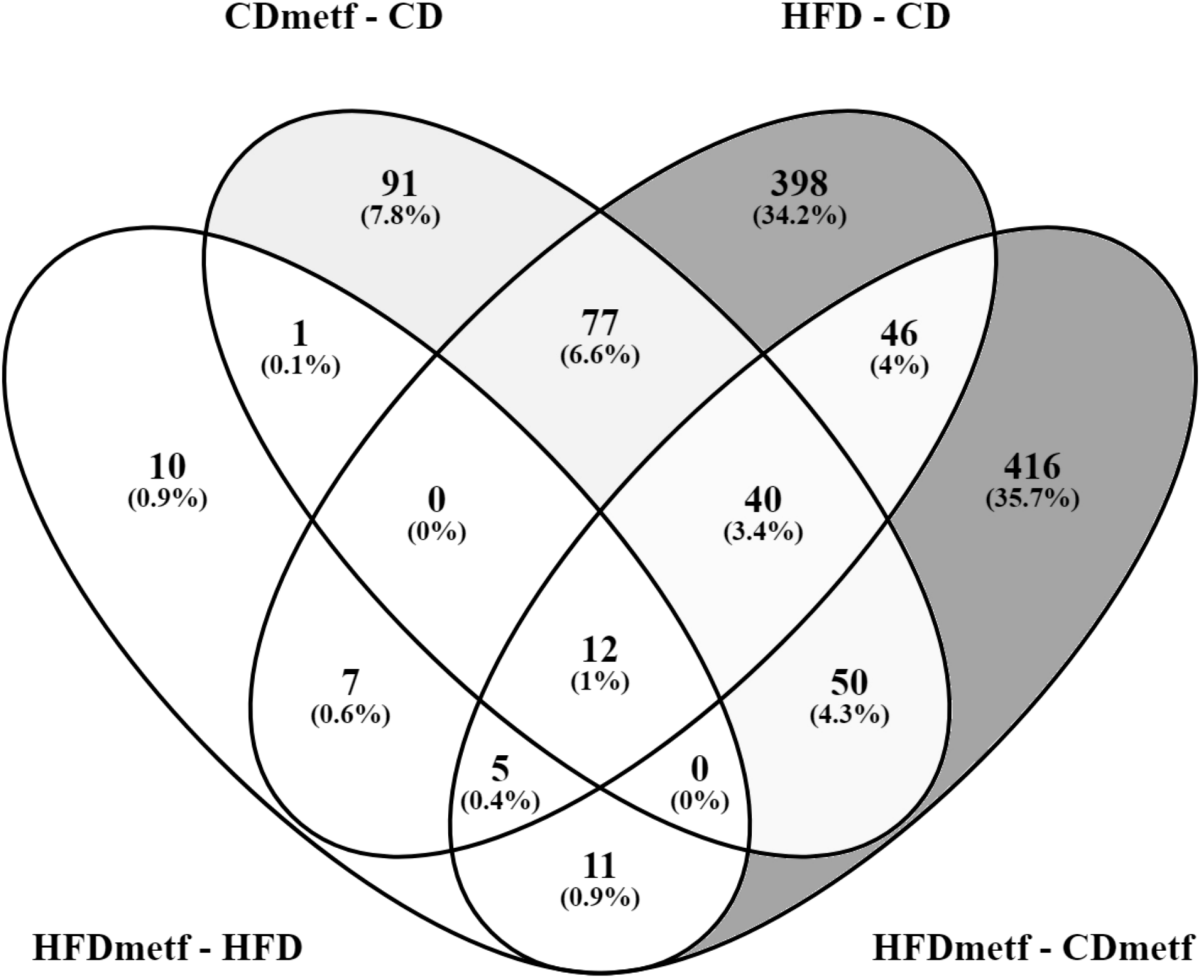
Venn diagram showing the distribution and overlap of differentially expressed genes among the analyzed contrasts. HFD, high-fat diet-fed animals; HFDmetf, high-fat diet-fed animals receiving metformin; CD, control diet-fed animals; CDmetf, control diet-fed animals receiving metformin.

To control for the effects of the diet, at first, the transcriptome profiles of animals receiving diets with distinct fat content (HFD vs. CD) and no metformin were compared, revealing an HFD-induced differential expression of 585 genes (FDR < 0.05). The majority of DEGs (395 genes) were uniquely differentially expressed in the HFD vs. CD contrast, including the ones functionally associated with lipid metabolism, such as *Bco1* (logFC = −3.08; FDR = 1.69E−03) and *Acaa1b* (logFC = 1.56; FDR = 2.07E−02) (**Table 1**). In total, 253 genes were downregulated, and 332 genes appeared to be upregulated due to increased dietary fat content solely (**Supplementary Table 1**, **Supplementary Figures 1, 2**). The comparison of the intestinal gene expression levels obtained from HFD-fed animals against CD-fed animals both receiving metformin indicated the modulatory effect of metformin on the diet-related intestinal transcriptome profiles exerted as the differential expression of 580 genes (333 downregulated and 247 upregulated). Out of those, 103 genes showed differential expression also in the HFD vs. CD contrast, while for the remaining majority (476 DEGs unique to the HFDmetf vs. CDmetf contrast), the diet-related alterations in expression levels were provoked directly by the presence of metformin (**Figure 3**, **Supplementary Table 3**, **Supplementary Figures 3, 4**).

Table 1List of the top 5 differentially expressed genes found in the distal part of the small intestine of animals in four different treatment- and diet-related contrasts, ranked by fold change.

**Table:** 

HFD vs. CD			
Gene symbol	Full name	logFC	FDR
*Cyp4a10*	Cytochrome P450, family 4, subfamily a, polypeptide 10	4.63	4.66E−03
*Igkv4-72*	Immunoglobulin kappa chain variable 4-72	4.49	4.78E−02
*Igkv3-7*	Immunoglobulin kappa variable 3-7	3.74	4.94E−02
*Ighv2-3*	Immunoglobulin heavy variable 2-3	3.74	3.77E−02
*Apoa4*	Apolipoprotein A-IV	3.53	3.31E−02

**Table:** 

HFDmetf vs. CDmetf			
Gene symbol	Full name	logFC	FDR
*Igkv2-109*	Immunoglobulin kappa variable 2-109	3.81	4.87E−02
*Fabp1*	Fatty acid binding protein 1, liver	3.64	4.52E−02
*Cyp4a10*	Cytochrome P450, family 4, subfamily a, polypeptide 10	3.24	1.86E−02
*Slamf6*	SLAM family member 6	−2.79	4.63E−02
*Igkv13-84*	Immunoglobulin kappa chain variable 13-84	2.73	1.86E−02

**Table:** 

HFDmetf vs. HFD			
Gene symbol	Full name	logFC	FDR
*Ighv1-7*	Immunoglobulin heavy variable V1-7	5.76	3.64E−02
*Slamf6*	SLAM family member 6	−2.79	4.63E−02
*Ifi209*	Interferon-activated gene 209	−2.39	4.63E−02
*Il27ra*	Interleukin 27 receptor, alpha	−2.38	3.64E−02
*Ly6d*	Lymphocyte antigen 6 complex, locus D	−2.28	4.63E−02

**Table:** 

CDmetf vs. CD			
Gene symbol	Full name	logFC	FDR
*Igkv4-72*	Immunoglobulin kappa chain variable 4-72	5.33	2.83E−02
*Ighv1-7*	Immunoglobulin heavy variable V1-7	5.09	7.13E−03
*Ighv5-16*	Immunoglobulin heavy variable 5-16	3.72	2.18E−02
*Ighv9-4*	Immunoglobulin heavy variable 9-4	3.41	4.12E−02
*Ighv1-58*	Immunoglobulin heavy variable 1-58	−3.24	8.09E−03

HFD, high-fat diet-fed animals; HFDmetf, high-fat diet-fed animals receiving metformin; CD, control diet-fed animals; CDmetf, control diet-fed animals receiving metformin.

The KEGG pathway analysis of the list of identified DEGs in the HFD vs. CD comparison showed significant enrichment of 29 pathways, including butanoate metabolism among the uniquely enriched pathways (not represented in other contrasts) (**Table 2**), while the GO analysis revealed the involvement of cellular response to the fatty acid and lipid metabolism exhibited by statistically significant enrichment of 197 GO terms in total. Additionally, 112 significantly enriched GO terms and 18 KEGG pathways were discovered by the functional annotation analysis of the DEGs identified in the comparison of both diet types together with metformin intervention (HFDmetf vs. CDmetf). Significant enrichment of 11 unique immunity-related signaling pathways was found representing the modulatory effects of metformin (**Table 2**, **Supplementary Tables 1, 3**).

Table 2List of the top 3 enriched KEGG pathways found in the distal part of the small intestine of animals in four different treatment- and diet-related contrasts, ranked by statistical significance.

**Table:** 

HFD vs. CD			
Pathway	Gene count	*P*-value	Genes
mmu04672:Intestinal immune network for IgA production	9	2.78E−05	*Ccl28*, *Ccr9*, *Cd28*, *Cd86*, *Cxcr4*, *H2-Ob*, *Itgb7*, *Madcam1*, *Tnfrsf13b*
mmu00900:Terpenoid backbone biosynthesis	7	4.41E−05	*Acat2*, *Hmgcs1*, *Hmgcs2*, *Idi1*, *Mvd*, *Mvk*, *Pmvk*
mmu04670:Leukocyte transendothelial migration	14	4.69E−05	*Ctnna3*, *Cxcr4*, *Icam1*, *Itgal*, *Mmp9*, *Ncf1*, *Ncf4*, *Pik3cd*, *Prkcb*, *Rac2*, *Rassf5*, *Sipa1*, *Txk*, *Vcam1*

**Table:** 

HFDmetf vs. CDmetf			
Pathway	Gene count	*P*-value	Genes
mmu05340:Primary immunodeficiency	7	4.57E−04	*Blnk*, *Btk*, *Cd3e*, *Cd79a*, *Il2rg*, *Lck*, *Tnfrsf13b*
mmu05168:Herpes simplex infection	17	5.01E−04	*Bmal1*, *H2-DMb2*, *H2-Ob*, *H2-T24*, *Hcfc2*, *Ifit1*, *Ifit1bl1*, *Irf7*, *Nfkbia*, *Oas1a*, *Pml*, *Rnasel*, *Skp2*, *Srsf2*, *Stat1*, *Stat2*, *Tlr9*
mmu04142:Lysosome	12	1.06E−03	*Acp5*, *Cd164*, *Gba*, *Gns*, *Lamp1*, *Lamp2*, *Naga*, *Napsa*, *Neu1*, *Slc11a1*, *Tcirg1*, *Tpp1*

**Table:** 

HFDmetf vs. HFD			
Pathway	Gene count	*P*-value	Genes
mmu05340:Primary immunodeficiency	2	6.85E−02	*Btk*, *Tnfrsf13b*
mmu04672:Intestinal immune network for IgA production	2	8.40E−02	*Cxcr4*, *Tnfrsf13b*
mmu04060:Cytokine-cytokine receptor interaction	3	8.86E−02	*Clcf1*, *Cxcr4*, *Tnfrsf13b*

**Table:** 

CDmetf vs. CD			
Pathway	Gene count	*P*-value	Genes
mmu04514:Cell adhesion molecules (CAMs)	12	8.79E−06	*Cd274*, *Cd28*, *Cd4*, *Cldn8*, *H2-M3*, *H2-Q6*, *Icam1*, *Itgal*, *Madcam1*, *Selplg*, *Spn*, *Vcam1*
mmu05340:Primary immunodeficiency	6	7.62E−05	*Blnk*, *Cd4*, *Cd79a*, *Il2rg*, *Tnfrsf13b*, *Zap70*
mmu04670:Leukocyte transendothelial migration	8	9.29E−04	*Cldn8*, *Cxcr4*, *Icam1*, *Itgal*, *Ncf1*, *Rassf5*, *Sipa1*, *Vcam1*

HFD, high-fat diet-fed animals; HFDmetf, high-fat diet-fed animals receiving metformin; CD, control diet-fed animals; CDmetf, control diet-fed animals receiving metformin.

### 3.3 Transcriptional effect of metformin

**Figure 4:**
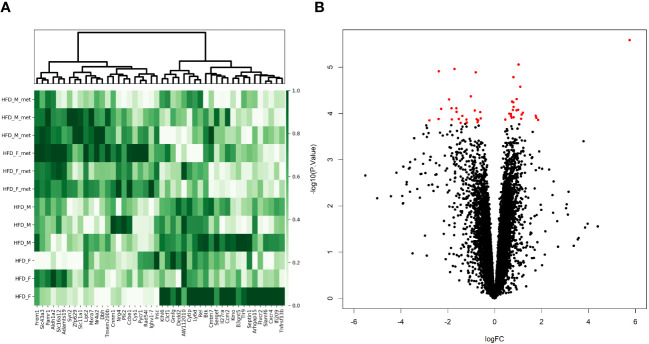
Metformin treatment-related alterations in gene expression profiles of the distal part of the small intestine in the high-fat diet-fed animal model. **(A)** Heatmap and hierarchical clustering of 46 DEGs identified. Each row corresponds to one animal and each column represents a DEG. Normalized sequence read counts were rescaled to lie in the range [0,1] and further used to estimate the difference between the gene expression levels in both experimental groups. DEGs with analogous expression values were clustered at the column level. **(B)** Volcano plot showing the distribution of gene expression comparing HFD-fed animals with and without metformin intervention. Statistical significance vs. log2 fold change is plotted on the *y*- and *x*-axes, respectively. The significant DEGs (FDR < 0.05) are shown as red dots, and black dots refer to genes with non-significant alterations in the expression levels.

**Figure 5:**
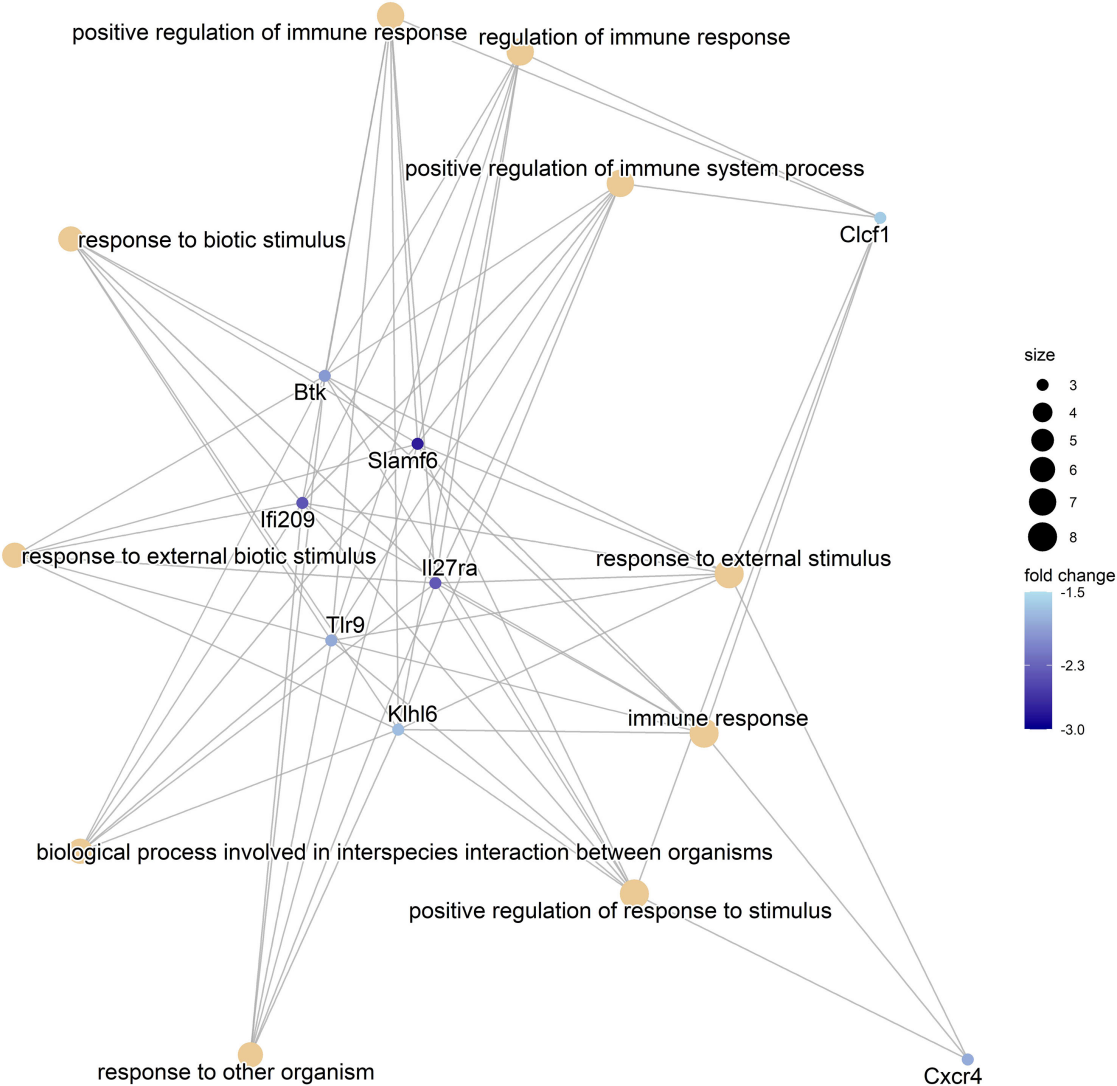
The Gene-Concept Network plot. The linkage between the 10 most significant GO terms and related genes identified in the comparison of HFD-fed animals receiving metformin and HFD-fed animals with no antidiabetic treatment (HFDmetf vs. HFD) is shown. The size of each node representing the Gene Ontology term is relative to the number of corresponding genes, and the intensity of the blue color reflects the fold change of the expression of the particular gene.

We considered the HFDmetf vs. HFD as the most representable contrast reflecting the host transcriptomic responses to metformin treatment in the presence of obesity and insulin resistance. The metformin-related comparison revealed apparent differences in the expression of 46 genes (23 genes downregulated and 23 genes upregulated in the intestinal tissue of metformin-treated mice), with the most prominent upregulation of the *ighv1-7* gene (logFC = 5.76; FDR = 3.64E−02) showing differential expression also in the contrast of CDmetf vs. CD (logFC = 5.09; FDR = 7.13E−03) (**Figures 4A, B**). The KEGG pathway analysis of 46 DEGs revealed the enrichment of three pathways: primary immunodeficiency, intestinal immune network for IgA production contrasts, and cytokine–cytokine receptor interaction (**Table 2**); nevertheless, none of these reached statistical significance, and all three pathways were targeted also in other contrasts. Eight significantly enriched GO terms were identified in total, all falling within the ontology group of biological processes, and the majority of them related to immune functions, e.g., adaptive immune response (**Supplementary Table 2**, **Figure 5**).

Similarly, the comparison of the intestinal transcriptome of CD-fed animals receiving metformin with equally fed animals not treated with metformin was done resulting in 271 DEGs (56 genes downregulated and 215 upregulated in metformin-treated animals), with 91 of the genes appearing exclusively in the particular contrast (CDmetf vs. CD), including *Ighv1-81* (logFC = 2.52; FDR = 7.13E−03) and *Ighv14-3* (logFC = 2.76; FDR = 8.09E−03) (**Figure 2**, **Supplementary Table 4**, **Supplementary Figures 5, 6**). The comparison of CDmetf vs. CD revealed 15 immunity-related KEGG pathways enriched, including leukocyte transendothelial migration, NF-kappa B signaling pathway, and the three pathways that were affected by metformin treatment in HFD-fed animals. In total, 98 GO terms were significantly enriched due to the administration of metformin in CD-fed animals with only two terms (adaptive immune response and positive regulation of interferon-gamma production) overlapping with the results of the HFD-fed animal analysis above (**Supplementary Table 4**).

### 3.4 The relationship between host gene expression and gut microbiome in response to metformin treatment

**Figure 6:**
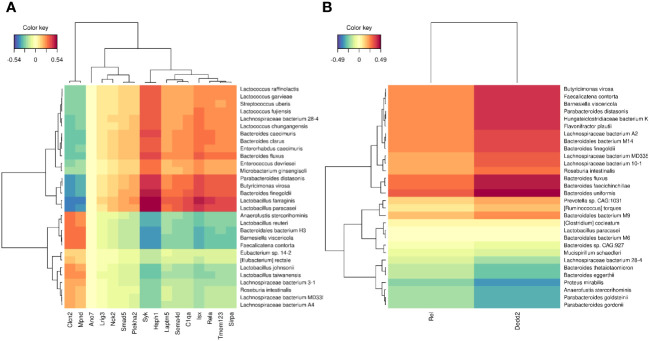
Heatmap of Pearson correlation coefficient representing the relationship between microbiota relative abundance and host intestinal gene expression levels. **(A)** Genes derived from the comparison of CDmetf vs. CD are shown. **(B)** DEGs of the HFDmetf vs. HFD contrast are shown. Up to 30 most discriminative features of both datasets are included according to the partial least squares discriminant analysis. Hierarchical clustering is applied to the genes and taxonomies according to the expression levels and abundance, respectively.

The compositional data of the animal gut microbiome were obtained at the end of the metformin intervention from the same animals and used for the integration with expression levels of DEGs identified in both contrasts reflecting the transcriptional effects of metformin (HFDmetf vs. HFD and CDmetf vs. CD). The correlation analysis of both datasets provided 31 metagenomics features of the highest relevance (VIP > 1) involving two genes from the comparison of HFD-fed animals and 16 genes derived from the contrast of CD-fed animals (**Figure 6**, **Supplementary Tables 6, 7**).

## 4 Discussion

Our data show a significant impact of metformin and a more prominent effect of the diet on the intestinal tissue gene expression profiles. Moreover, we report a property of metformin to modify the intestinal transcriptional effects of the diet with altered fat content and highlight the modulatory effect of the drug on mucosal immunity in both HFD- and CD-fed animals. To the best of our knowledge, this is the first study providing bulk RNA-Seq data of the small intestine obtained from a metformin-treated animal model of obesity and insulin resistance of both sexes *in vivo*.

In the comparisons of HFDmetf vs. HFD and HFDmetf vs. CDmetf, a GO term named positive regulation of I-kappa B kinase/NF-kappa B signaling appeared among the significantly enriched ontologies with a notable overlap of ontology-related genes between both contrasts showing the same direction of differential expression (*Plk2*, *Rel*, *Tlr9*). Moreover, the NF-kappa B signaling pathway was significantly enriched in the KEGG pathway analysis, particularly in the comparisons of CDmetf vs. CD and HFDmetf vs. CDmetf, with a conforming direction of up to nine pathway-associated gene expressions. Our study indicates the ability of metformin to downregulate the particular pathway in the distal part of the small intestine of healthy mice with an even more prominent effect in the presence of a diet with a relatively high fat content. These data are in line with previous *in-vitro* and *in-vivo* evidence of the ability of metformin to reduce NF-κB activation in the context of breast cancer stem cell growth (28) and to attenuate the hyperglycemia-induced activation of TLR/NF-κB/TNF-α/CXCL1/KC signaling pathway in the skeletal muscle of diabetic rats (29). In addition, correlation analysis of the gut microbiome-derived taxon relative abundance and expression levels of the DEGs appearing in both metformin-related contrasts revealed a positive correlation between two genes, namely, *Rel* and *Rela*, both subunits of NF-kappa B transcription complex (30, 31). The expression of the *Rela* gene correlated with the abundance of two representatives of the *Lactobacillus* genus, namely, *L. farraginis* and *L. paracasei*, both showing increased levels due to metformin treatment in mice before (9) and also with a short-chain fatty acid-producing *Parabacteroides distasonis*, which has been previously linked with metformin-related glucose modulatory effects (32). Meanwhile, the *Rel* gene expression correlated with the levels of *Bacteroides* spp. including *B. uniformis*, which has been associated with improved metabolic and immune functions in obese mice before (33). Since NF-κB family transcriptional factors are among the key regulators of host responses to microbial infection with a crucial role in gut homeostasis (34), our data indicate that the NF-kappa B signaling pathway may be targeted by metformin-induced alterations in the composition of the gut microbiome although substantiation of such a hypothesis should be done by additional studies. We believe that our study provides additional information about the possible molecular targets underlying the negative effect on the NF-κB activation as well as proves the existence of the same concept in the small intestines of mice even with normal weight and insulin sensitivity.

The enrichment of the primary immunodeficiency pathway was observed as a result of metformin treatment in both HFD- and CD-fed animals. Although there is no clinical evidence of the beneficial effect of metformin in primary immunodeficiency diseases reported so far, several studies show that metformin improves B-cell function and antibody production, particularly in type 2 diabetes mellitus patients reversing the hyperglycemia-induced damage (35, 36). Previous studies are not focused on the identification of specific molecular targets triggering improvements in humoral immunity although our data show several potential key genes for this interaction, such as *Blnk* coding for the B-cell linker protein, essential in the B-cell development and B-cell antigen receptor signaling pathway (37), and *Btk* gene coding for Bruton’s tyrosine kinase involved in pre-B-cell receptor signaling, survival of immature B cells in the bone marrow, and the development of peripheral B cells, both representing the primary immunodeficiency pathway (38). The modulatory effect of metformin on humoral immune responses is also supported by the differential expression of the *Tnfrsf13b* gene coding for TNF receptor superfamily member 13B. The *Tnfrsf13b* human homolog gene is coding for the TACI protein, which is essential in class-switch recombination, plasma cell differentiation, and antibody secretion (39). Interestingly, *tnfrsf13B* also maintains the secretion of intestinal sIgA in a T-cell-independent manner (40). Although our experiment revealed reduced levels of fecal sIgA in metformin-treated animals contrary to observations made in humans before (15), the RNA-Seq data, representing animal-derived transcriptome profiles with *tnfrsf13B* and immunoglobulin heavy-chain variable region coding gene *Ighv1-7* among the top hits, are in line with the previous hypothesis of metformin shifting the intestinal immunoglobulin responses (14). The discrepancies between human and mice data in the observed metformin-transformed sIgA levels may be caused by several overlooked sIgA-modulating factors such as aging itself (41) or another site of metformin action since previous reports show that the number of IgA-positive B cells is reduced due to the HFD in mice particularly in the colon but not in the small intestine (14). Moreover, the clinically relevant metformin dose applied in the particular study (50 mg/kg/day) may not be large enough to initiate drastic changes in IgA secretion.

Another interesting finding was the differential expression of the *Cxcr4* gene, coding for C-X-C Motif Chemokine Receptor 4, responsible for angiogenesis and metastasis of cancer (42) as well as the intestinal epithelial barrier maturation and restitution (43). Moreover, Cxcr4 and IgG-expressing plasma cells are even considered a marker of intestinal inflammation in humans (44). The significant metformin-induced downregulation of the *Cxcr4* gene was observed in HFD-fed animals (logFC = −1.83; FDR = 4.63E−02), while the comparison of HFD vs. CD with no metformin intervention showed upregulation of the gene (logFC = 1.43; FDR = 2.44E−02), suggesting that metformin reverses the HFD-related upregulation of the *Cxcr4* gene. Previous studies show that metformin downregulates the CXCL12/CXCR4 signaling in breast cancer and prostate cancer cell lines (45, 46); nevertheless, no data were supporting this pleiotropic effect of metformin in the small intestine so far.

Several limitations of this experiment should be considered in future studies. Firstly, one may consider expanding the number of animals to acquire more robust observations since there was a notable heterogeneity in both phenotypic measures and the observed intestinal transcriptome profiles among the study animals most probably due to several inevitable biases such as increased stress levels in co-housed males and estrous cycle in females. In addition, although both rodent diets contained similar ingredients except for the fat content, using identical food additives such as colorants may reduce the potential bias in the comparisons between both diet-related treatment arms. Exploring other intestinal sections such as the colon may reveal additional molecular targets of metformin, while focusing particularly on isolated Payer’s patches may provide knowledge about the mechanism of interaction between metformin and sIgA. Finally, the absence of hyperglycemia in several study animals of the HFD group restricted the ability to draw strong conclusions about the diabetes-specific effects of the drug.

In summary, the present study provides a complex dataset reflecting both diet- and metformin-induced gene expression alterations in the ileum of C57BL/6N mice showing the overwhelming dominance of the diet fat content-related transcriptional footprint. In addition, our data support the immunomodulatory effect of metformin with significant enrichment of previously described molecular targets of the drug, such as the downregulation of the NF-kappa B signaling pathway in previously unrecognized tissue and a prominent emphasis on the genes involved in gut humoral responses.

## Data availability statement

The data presented in the study are deposited in the Gene Expression Omnibus repository, accession number GSE240206.

## Ethics statement

The animal study was approved by National animal welfare and ethics committee, State Food and Veterinary Service (Approval No. Nr.1.1-13/17/1745). The study was conducted in accordance with the local legislation and institutional requirements.

## Author contributions

MB, LS, and IK performed the animal experiment. MB, LA, and LB performed the sample analysis. MB and IS performed the data analysis and interpretation. MB and LJ drafted the manuscript. JK and IE provided critical revision of the manuscript. MB and IE collaborated in funding acquisition. JK performed supervision and conceptualization of the study. All authors contributed to the article and approved the submitted version.
