# Supplementary material for: Metformin targets intestinal immune system signaling pathways in a high-fat diet-induced mouse model of obesity and insulin resistance
Source: Front Endocrinol (Lausanne). 2023 Sep 19;14:1232143. doi: 10.3389/fendo.2023.1232143 (PMC10546317; doi:10.3389/fendo.2023.1232143)
Supplement: Supplementary file 8 [file Image_1.pdf]

Supplementary Figures

Metformin Targets Intestinal Immune System Signaling Pathways in High-Fat Diet-Induced Type 2 Diabetes Mouse Model of Obesity and Insulin Resistance

Monta Brīvība[1]*, Laila Silamiķele[1], Ineta Kalniņa[1], Ivars Silamiķelis[1], Līga Birzniece[1], Laura Ansone[1], Lauma Jagare[1], Ilze Elbere[1], Jānis Kloviņš[1]

*** Correspondence:** Corresponding Author: monta@biomed.lu.lv

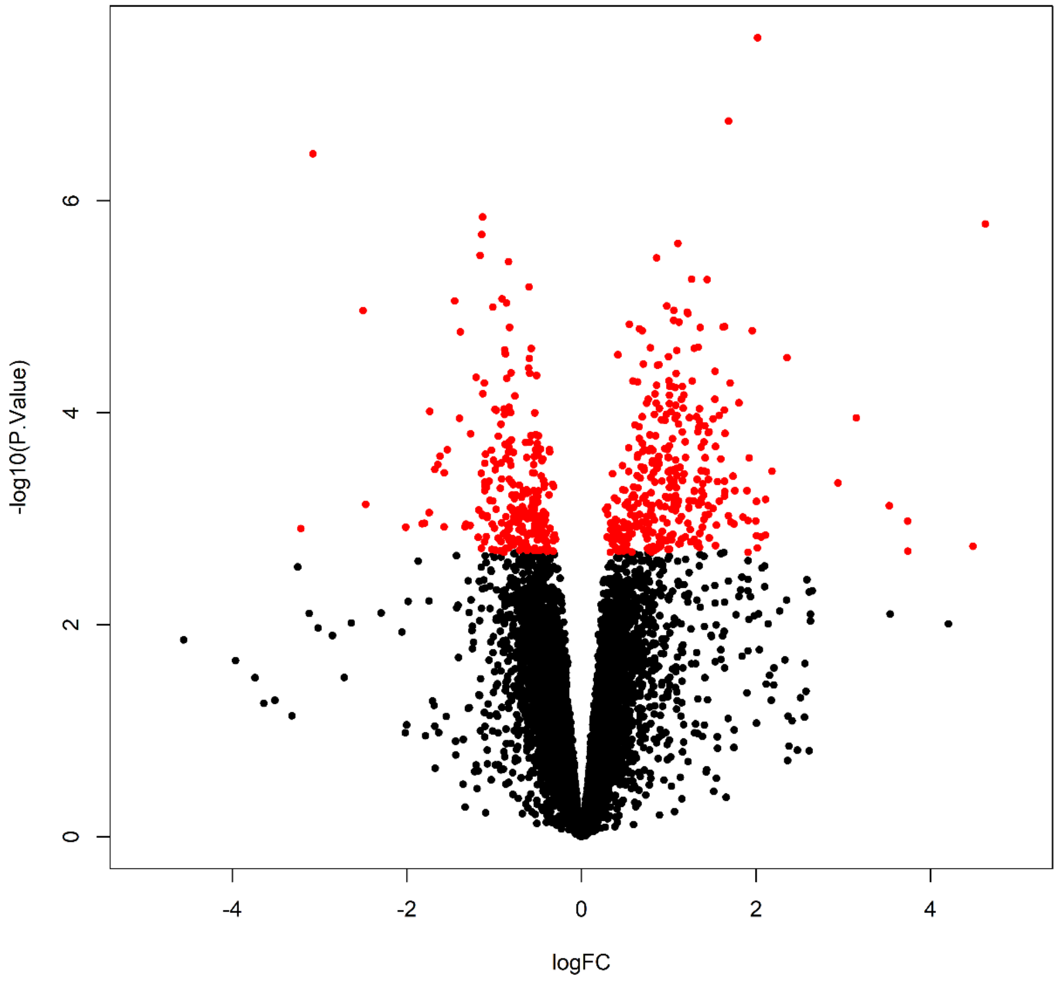

Supplementary Figure 1. Volcano plot showing the distribution of gene expression comparing high-fat diet-fed animals with control diet-fed animals and no therapeutic intervention. Statistical significance versus log2 fold change is plotted on the y and x axes, respectively. The significant DEGs (FDR < 0.05) are shown as red dots, and black dots correspond to genes with nonsignificant alterations in the expression levels.

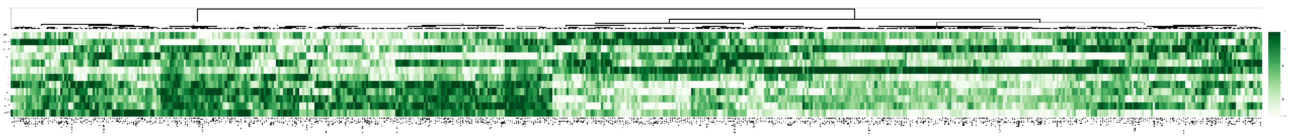

Supplementary Figure 2. Heat map and hierarchical clustering of 585 DEGs identified comparing high-fat diet-fed animals with control diet-fed animals and no therapeutic intervention. Each row corresponds to one animal and each column represents a DEG. Normalized sequence read counts were rescaled to lie in the range [0,1] and further used to estimate the difference between the gene expression levels in both experimental groups. DEGs with analogous expression values were clustered at the column level.

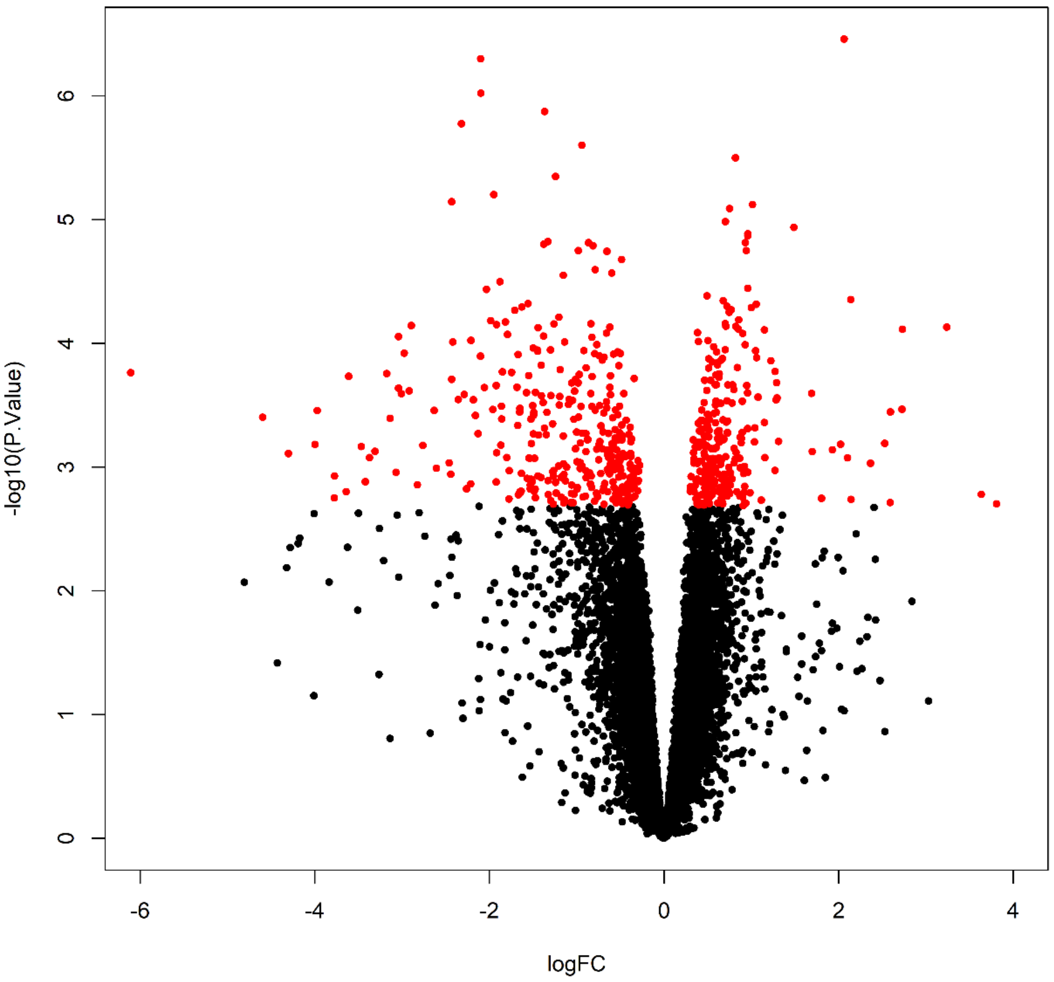

Supplementary Figure 3. Volcano plot showing the distribution of gene expression comparing high-fat diet-fed animals with control diet-fed animals both receiving metformin. Statistical significance versus log2 fold change is plotted on the y and x axes, respectively. The significant DEGs (FDR < 0.05) are shown as red dots, and black dots correspond to genes with nonsignificant alterations in the expression levels.

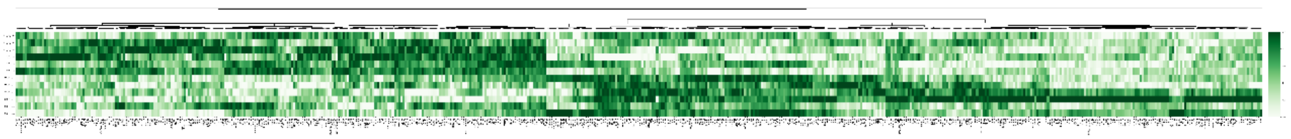

Supplementary Figure 4. Heat map and hierarchical clustering of 580 DEGs identified comparing high-fat diet-fed animals with control diet-fed animals both receiving metformin. Each row corresponds to one animal and each column represents a DEG. Normalized sequence read counts were rescaled to lie in the range [0,1] and further used to estimate the difference between the gene expression levels in both experimental groups. DEGs with analogous expression values were clustered at the column level.

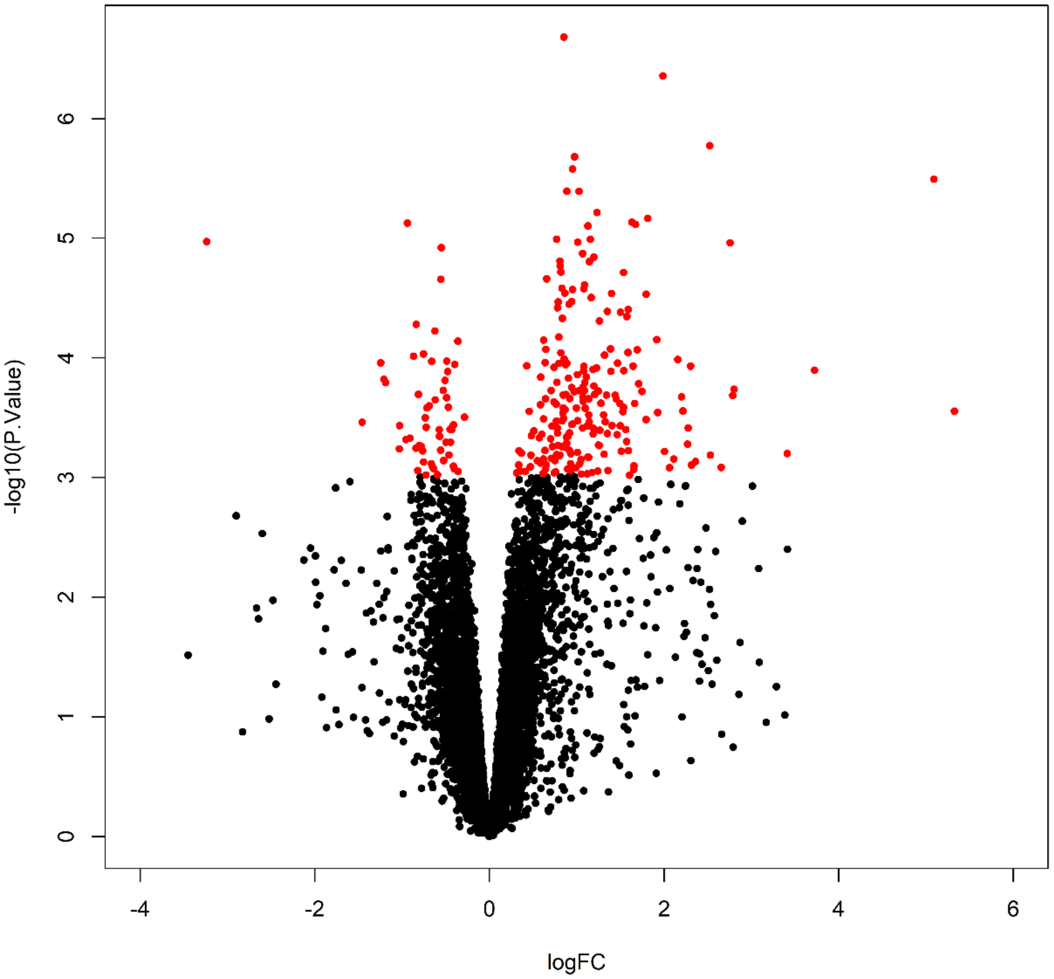

Supplementary Figure 5. Volcano plot showing the distribution of gene expression comparing control diet-fed animals receiving metformin with control diet-fed animals not receiving metformin. Statistical significance versus log2 fold change is plotted on the y and x axes, respectively. The significant DEGs (FDR < 0.05) are shown as red dots, and black dots correspond to genes with nonsignificant alterations in the expression levels.

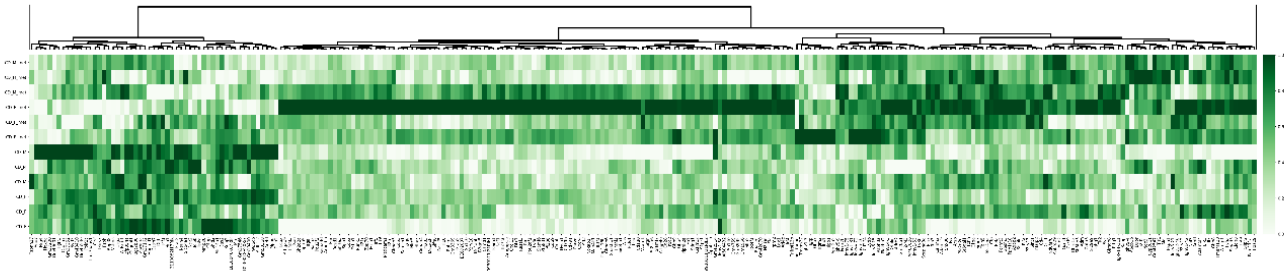

Supplementary Figure 6. Heat map and hierarchical clustering of 271 DEGs identified comparing control diet-fed animals receiving metformin with control diet-fed animals not receiving metformin. Each row corresponds to one animal and each column represents a DEG. Normalized sequence read counts were rescaled to lie in the range [0,1] and further used to estimate the difference between the gene expression levels in both experimental groups. DEGs with analogous expression values were clustered at the column level.

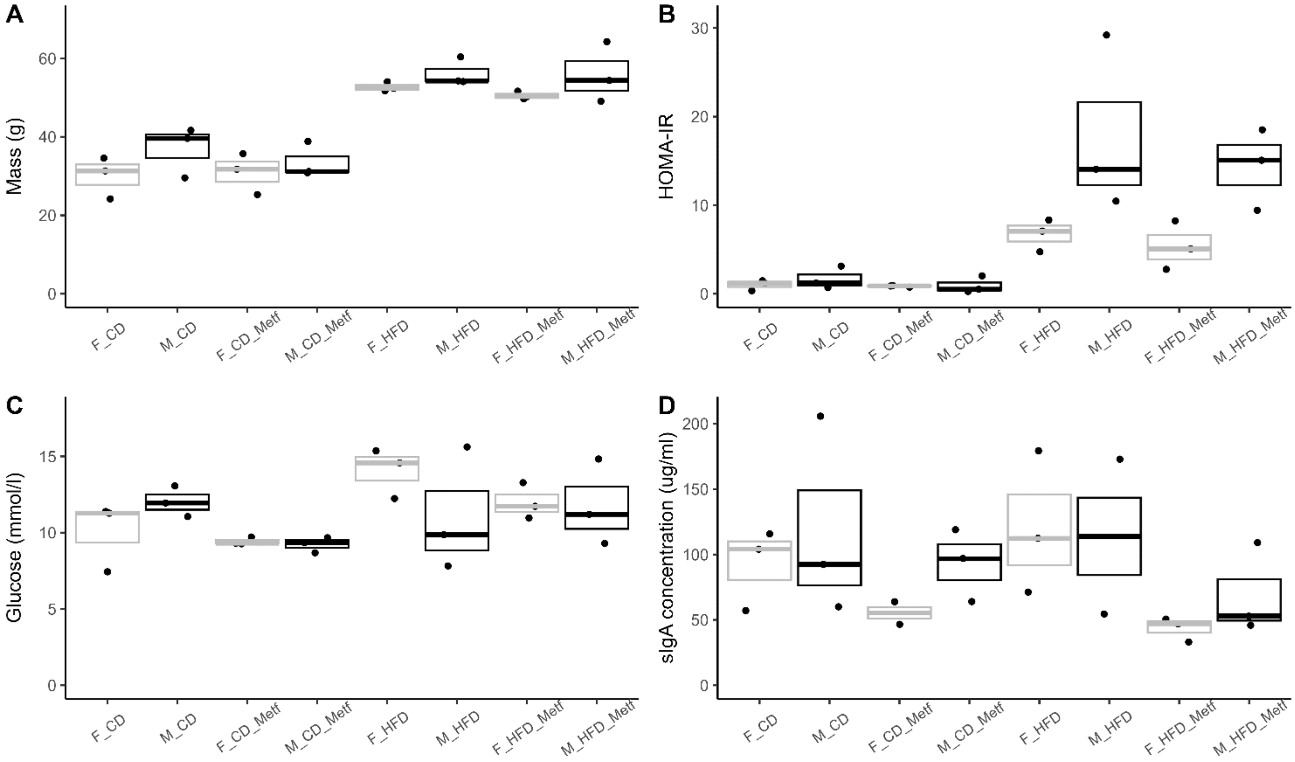

Supplementary Figure 7. Jitter plots representing the phenotypic differences among animals of different treatment arms at the end of the study stratified by sex. Four different measurements are shown: **(A)** body mass, **(B)** Homeostatic Model Assessment for Insulin Resistance, **(C)** blood glucose levels, and **(D)** Secretory IgA levels. Each dot represents a single measurement for each animal with grey crossbars indicating the median and IQR of female animals of the corresponding experimental group, while the black crossbars show the median and IQR of male animals of the corresponding experimental group. F_CD: control diet-fed female animals; F_CD_Metf: control diet-fed female animals receiving metformin; F_HFD: high-fat diet-fed female animals; F_HFD_Metf: high-fat diet-fed female animals receiving metformin, M_CD: control diet-fed male animals; M_CD_Metf: control diet-fed male animals receiving metformin; M_HFD: high-fat diet-fed male animals; M_HFD_Metf: high-fat diet-fed male animals receiving metformin, IQR: interquartile range.
